# Minimum acceptable diet and associated factors among 6–23 months children in rural households with irrigated users and non-irrigated users in Ethiopia: a comparative cross-sectional study

**DOI:** 10.1186/s40795-024-00873-7

**Published:** 2024-04-19

**Authors:** Welelaw Mengistu, Dereje Birhanu, Omer Seid

**Affiliations:** 1 Ambomesk Health Center, North Mecha Health Office, West Gojjam Health Department, West Gojjam, Ethiopia; 2 Department of Nutrition and Dietetics, School of Public Health, College of Medicine and Health Sciences, Bahir Dar University, Bahir Dar, Ethiopia

**Keywords:** Minimum acceptable diet, Irrigated and non-irrigated users, Mecha district

## Abstract

**Introduction:**

Poor quality of complementary foods is a key contributor to undernutrition even with optimal breastfeeding. Minimum Acceptable Diet (MAD) has tremendous health and nutrition benefits but only 12% of Ethiopian children’s feeding practices meet its standards. The Ethiopian government has recently increased efforts to expand nutrition-sensitive irrigation to improve child nutrition. However, the impact that irrigation has brought on the minimum acceptable diet practice of children below two years has not yet been studied. The aim of this study was to compare the magnitude of MAD practice and associated factors among children aged 6–23 months in households with irrigated users and non-users of North Mecha district, Ethiopia.

**Methods:**

A community-based comparative cross-sectional study was employed among 824 mother-child pairs. For infant and young child feeding practices, the data collection tools were adapted from the World Health Organization’s standardized questionnaire developed in 2010. X^2^ test was used to compare the MAD practices of irrigated users’ and non-irrigated users. Bivariate and multivariable logistic regression analyses were performed to see the predictor variables. p-value < 0.05 was taken to declare statistical significance.

**Results:**

The present study showed that the MAD practice of under two children in irrigated users is significantly higher than non-users (X^2^ = 13.91, *P* <.001). Maternal involvement in decision-making [AOR = 4.37, 95% CI: (2.05,9.33)], initiation of breastfeeding [AOR = 5.29, 95% CI: (2.393,11.672)], and history of illness [AOR = 4.10, 95%CI: (1.48,11.38)] were independent predictors for MAD practice among irrigated users. Whereas, maternal involvement in decision making [AOR = 4.71, 95% CI: ( 2.28, 9.75)], place of delivery [AOR = 2.51, 95% CI: ( 1.14, 5.55)], postnatal care (PNC) follow-up [AOR = 3.01, 95%CI: (1.57, 5.77)] and growth monitoring and promotion (GMP) service utilization [AOR = 4.64, 95% CI: (2.40, 8.95)] were the independent predictors among the non-users.

**Conclusion:**

MAD practice was much higher in irrigated users than in non-irrigated users. Involvement in a decision, place of delivery, PNC, and GMP are independent predictors of MAD in children from non-irrigated households. The study suggested that expanding access to irrigation to households may be the best approach to improve child nutrition.

## Introduction

Practices related to infant and young child feeding (IYCF) have a direct or indirect influence on the development, health, and nutritional status of children under the age of two, which ultimately affects the survival of the child [1–3]. World Health Organization (WHO) recommends that infants be exclusively breastfed for the first six months of life to achieve optimal growth, development, and health. Thereafter, to meet their evolving nutritional needs, infants should receive solid, semi-solid, and soft complementary foods while breastfeeding continues for up to two years of age or beyond. After the first six months of life, infants’ nutrient demands start to exceed what breast milk alone can provide, and this leaves them vulnerable to malnutrition unless appropriate complementary foods are introduced [4, 5].

Complementary foods need to be nutritionally adequate, safe, and appropriately fed in order to meet the child’s energy and nutrient needs. Minimum Acceptable Diet (MAD) is an IYCF indicator designed to measure the appropriate complementary feeding patterns of children aged 6–23 months. Minimum dietary diversity (MDD) and minimum meal frequency (MMF) are the components of the MAD composite indicator, which is used to quantify food intake for children who are breastfed, and for non-breastfeeding children, MDD, MMF, and minimum milk feeding frequency (MMFF). The indicator provides a useful way to track progress in simultaneously improving the key quality and quantity dimensions of children’s diets [6, 7].

The quality and quantity of a child’s diet are more important before age two than at any other time in life [8, 9]. A minimum acceptable diet is essential to ensure appropriate growth and development for infants and children aged 6–23 months. Without adequate diversity and meal frequency, infants and young children are vulnerable to malnutrition, especially stunting and micronutrient deficiencies, and to increased morbidity and mortality [10]. Diet quality is associated with nutrition status; children who are fed at least a minimum acceptable diet are less likely to be stunted or underweight [11–13]. Research shows that appropriate complementary feeding has the potential to prevent 6% of all under-five deaths, particularly in developing countries (9). Unhygienic feeding practices also increase the risk of infections and diarrhoea in young children, which, when combined with poor diets, can lead to growth failure [9, 14].

Despite widespread consensus on the importance of good nutrition in early life, an alarming number of young children are practicing poor complementary foods [15, 16]. The most recent global estimates of complementary feeding practices based on indicators established by the WHO highlight a worrying situation. In low-and middle-income countries, half of all children are not receiving the minimum meal frequency (the minimum number of meals throughout the day needed to meet their nutrient needs); more than two-thirds of children are not receiving the minimum dietary diversity (meals from a minimum number of food groups); and five out of six children are not receiving a minimum acceptable diet (both the minimum meal frequency and minimum dietary diversity needed to reduce the risk of malnutrition) [17].

In Ethiopia, children have some of the lowest dietary diversity rates in the world. Their diet is often lacking in animal-source foods, fruit, and vegetables. The resent estimate showed that only 7% of Ethiopian children aged 6–23 months met the MAD standards (with MMF at 45% and MDD at 14%). The Amhara region has a low rate of MAD standards (only 3% of children aged 6–23 months met the MAD standards). In the country, significant number of young children are suffering the consequences of poor diets [18]. Stunting affects 36.8% of children under five, diminishing their physical and cognitive growth and development [3]. Children affected by stunting often grow up to be stunted adults themselves, and stunted mothers are more likely to have stunted children. Indeed, undernutrition is responsible for up to 50% of deaths in children under 5 and is a significant cause of morbidity in this age group [19].

The Ethiopian government has designed a multi-sectoral plan of nutrition intervention that aims to address the immediate, underlying, and basic causes of malnutrition to end child undernutrition by 2030 [20]. The Ministry of agriculture (MoA) is one of the sectors working on year-round availability, access, and consumption of variety, safe, and nutritious foods, which helps to address the underlying determinants of infant and young child nutrition [21–23]. As its part, MoA has been working to increase access to small scale irrigated households to improve year-round diversified production to improve both the quality and quantity of the diets of rural households [21, 24]. However, the impact that irrigation has brought on the minimum acceptable diet practice of children under two years old has not yet been studied. Therefore, this study was designed to compare minimum acceptable diet practice and associated factors among children aged 6–23 months in households with irrigated and non-irrigated users in North Mecha district, Northwest Ethiopia. The study examined the role of irrigation in improving the MAD practices of children.

## Methods and materials

### Study area

The study was carried out in the Amhara region, North Mecha district, which is 34 km southwest of Bahir Dar, the hub of the Amhara National Regional State, and 530 km northwest of Ethiopia’s capital, Addis Ababa. The district’s expected population in 2021 was 317,885, with 157,353 men and 160,532 women, according to the results of the 2007 national census [25]. The district comprises three climatic zones: highland “Dega”, mid-altitude “Weyna Dega” and lowlands “Kola”. The mean annual rainfall ranges from 1,000 mm to 2,000 mm. The district has 156,027 hectares of area, of which 72,178 hectares were used for cultivation and about 1,386 hectares were covered by water bodies. There are 37 rural Kebeles among these 10 Kebeles irrigated users, and the remaining are non-irrigated users. Maize and millet are mainly planted during the main rainy season, while wheat, maize, and vegetables are grown under irrigated conditions [26, 27].

### Study design and period

A Community-based comparative cross-sectional study design was employed to assess minimum acceptable diet practice and its associated factors among children aged 6–59 months from irrigated and non-irrigated households in Mecha district. The study was conducted from September to October, 2021.

### Population

All children aged 6–23 months in both irrigation and non-irrigated users in the district were the source population for the study. All children aged 6–59 months in the randomly selected kebele (for both irrigation and non-irrigated) were the study population. Mother/care takers were interviewed for the study.

### Eligibility criteria

All children aged 6–23 months who had been leaving at least for 6 months in the selected irrigated and non-irrigated user kebeles were included in the study.

### Study variables

Minimum acceptable diet practice, which is a combination of minimum meal frequency and minimum dietary diversity for breastfeeding and non-breastfeeding children, is the outcome (dependent variable) variable for the study. Whereas, socio-demographic and economic, maternal, and child related factors were included in the study.

### Sample size determination

As it was a comparative cross-sectional study, the minimum sample size was determined by using the double population proportion formula [n (in each group) = f (α, β) (p1q1 + p2q2) / (p1 - p2) ²], with the following assumptions that the two groups were considered based on their irrigation status. Group one was without irrigation as not exposed (non-user HHs), and group two was with irrigation as exposed (user HH). To estimate the minimum sample size of the study, 35.5% of households with non-irrigated users attain minimum acceptable diet practice among mothers having children aged 6–23 months in Mereka district, Southern Ethiopia [28], and households practice from irrigation users not known, so 50% (*P* =.5) were taken. Then, the sample size estimation was calculated using Epi Info software, and with a 95% confidence level and an 80% power yielded 392, multiplied by a design effect of 2, and then adding a 5% non-response rate, the final required sample size was 824.

### Sampling procedure

**Fig. 1:**
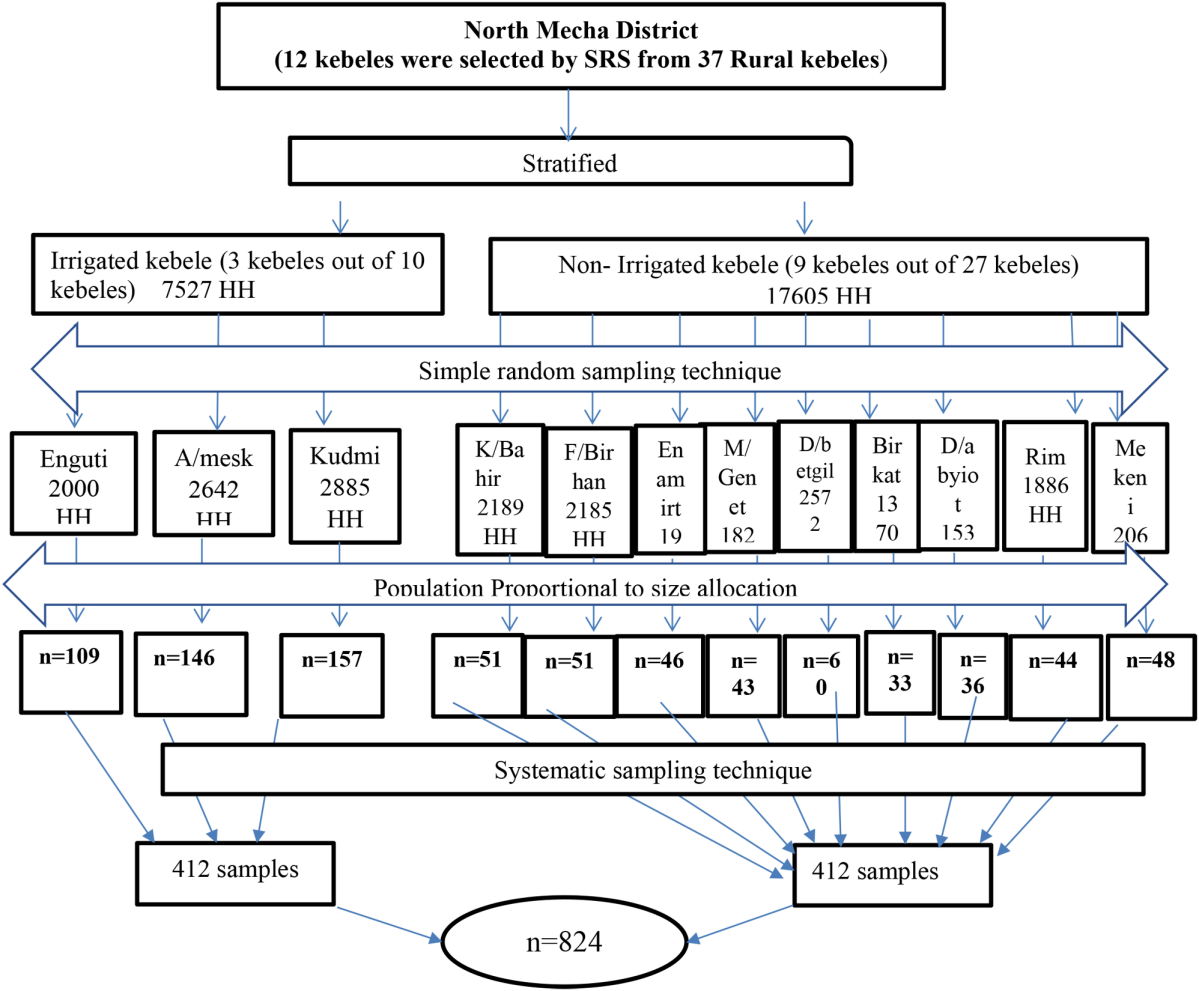
Sampling procedure to study minimum acceptable diet and associated factors among infant and young children aged 6–23 months in irrigated and non-irrigated areas of north Mecha district, northwest Ethiopia, 2021

A stratified sampling technique was used to select the study population. In the first stage, rural kebeles were stratified by irrigation land use as irrigated and non-irrigated users. In the next stage, a total of twelve kebeles (three from irrigated and nine from non-irrigated kebeles) were selected randomly using the lottery method. After allocating a sample size to each kebele of the HHs with irrigated and non-irrigated users, the required sample size was selected using a systematic sampling technique (Fig. 1). A single child was selected by the lottery method from the households in which two or more 6- to 23-month-old months children were found.

### Operational definition of terms

#### Minimum acceptable diet

The percentage of breastfed children 6–23 months of age who had at least the minimum dietary diversity and the minimum meal frequency during the previous day and non-breastfed children 6–23 months of age who received at least two milk feedings and had at least the minimum dietary diversity, not including milk feeds, and the minimum meal frequency during the previous day [4, 29].

#### Minimum dietary diversity

Percentage of children 6–23 months of age who consumed foods and beverages from at least five out of eight defined food groups during the previous day. The eight food groups used for tabulation of this indicator are: breast milk; grains, roots, tubers and plantains; pulses (beans, peas, lentils), nuts and seeds; dairy products (milk, infant formula, yogurt, cheese); flesh foods (meat, fish, poultry, organ meats); eggs; vitamin-A rich fruits and vegetables; and other fruits and vegetables [6].

#### Minimum meal frequency

A child receives solid, semi-solid, or soft foods (but also includes milk for non-breastfed children) the minimum number of times or more over the previous day. The minimum number of times is 2 times for breastfed infants 6–8 months, 3 times for breastfed children 9–23 months, and 4 times for non-breastfed children 6–23 months in the last 24 h [6, 29].

#### Food insecurity

It is defined as a situation where people, individuals at times, lack physical and economic access to sufficient, safe, and nutritious food needed to maintain a healthy and active life. Household food security was assessed using the Household Food Insecurity Access Scale validated tool developed by Food and Nutrition Technical Assistance. The tool has nine occurrence questions followed by nine frequency of occurrence questions that measure the severity of household food insecurity in the past 4 weeks [30].

#### Household wealth index

A proxy measure of living standards derived from information on ownership available assets and household characteristics and households classified into five categories [18].

#### Irrigation

It is defined as the application of artificial water to the living plants for food production and overcoming the shortage of rainfall and helping to stabilize agricultural production and productivity [31]. Irrigation users are households who did use irrigation land whereas those households who did not use irrigation land were called “non-users”.

#### Maternal knowledge on IYCF practice

Knowledge of mothers on infant and child feeding practice was measured based on ten knowledge questions. Each correct answer (yes) earned one point, and any wrong answer (no) got zero. The calculated knowledge score ranged from 0 to 10 points. Those who score above the mean (5.7 ± 2.6 standard deviations) was categorized as knowledgeable and those who score below the mean was categorized as not knowledgeable.

#### Data collection tool and procedure

Mothers with children between the ages of 6 and 23 months were interviewed in-person during a home visitation, and information was gathered using a semi-structured questionnaire. The questionnaire was adopted from different literatures. The data collection tool consists of six parts; socio-demographic, wealth index, maternal and child health, maternal knowledge of IYCF practice, 24-hour recall child feeding practice, and household food insecurity. The socio-demographic tools were adapted from the 2016 Ethiopia Demography and Health Survey (EDHS) [18]. The child’s dietary diversity was assessed using the 24-hours dietary recall method [6]. Wealth status was assessed by using questions adapted from the 2016 Ethiopian Demographic and Health Survey (EDHS) and other literature [18, 32]. Household food insecurity was assessed using Household Food Insecurity Access Scale validated tool (HFIAS) [33].

#### Data quality assurance

To maintain consistency, questionnaires were written in English, translated into the Amharic language spoken there, and then back into English. Both the supervisor and the data collectors received a three-day training on the goals of the research and the general methods of data collection producer. Before the actual data collection, a pretest including 5% of the samples in comparable locations was carried out. The principal investigators and supervisors verified the consistency and completeness of the gathered data every day.

#### Data management and analysis

Data were entered using Epi Data entry client version 4.6.0.6 and exported to SPSS 23.0 statistical package for analysis. Data cleaning was performed to check for consistency and values. A dietary diversity score was computed out of eight food groups, and household economic status was measured by constructing a wealth index. After labeling the variables between 0 and 1, the Principal Component Analysis (PCA) was applied. Then, the wealth status is ranked and labeled as richest, rich, middle, poorer, and poorest from the highest to the lowest rank. The HFIAS indicator categorizes households into four levels of household food insecurity (access): food-secure, mild, moderately, and severely food insecure ( < = 1, 2–10, 11–17, and > 17) respectively. A bivariable logistic regression analysis was done to assess the association between each covariate with MAD. Covariates with p value less than 0.2 during bivariable logistic regression analysis were included in a multivariable logistic regression model to control all possible confounders and identify factors significantly associated with MAD. Unadjusted and adjusted odds ratios along with a 95% confidence interval was estimated to assess the strength of the association of each explanatory variable with MAD. Variables with a p value < 0.05 in the final model were declared statistically significant. Hosmer-Lemeshow goodness-of-fit was used to test the model’s fitness.

## Results

### Socio-demographic and economic characteristics

Among the 824 sampled subjects, 775 children aged between 6 and 23 months with mothers/care givers were enrolled in the study making a response rate of 98% (94.4% in irrigation users and 93.7% in non-irrigation users). Among children aged 6–23 months, 189 (48.6%) and 204 (52.2%) were male by sex from irrigation users and non-irrigation users, respectively. The mean age of mothers was 30.3 (± 6.8) years for irrigated users and 30.3 (± 6.7) years, for non-irrigated users, respectively. All study subjects, both irrigation users and non-irrigation users, were orthodox Christians by religion and Amhara by ethnicity. One hundred eighty-nine (48.6%) mothers among the irrigation users and 189 (49%) among non-irrigation users were involved in household decision-making (Table 1).

**Table 1:** Socio-demographic and economic characteristics of the respondents from irrigation users and non-users of North Mecha District, Northwest, Ethiopia, 2021 (N1 = 386, N2= 389)

Characteristics		Irrigation			
		Non-users (N1=386)		Users (N2=389)	
		Frequency	%	Frequency	%
Sex of the child	Male	204	52.8	189	48.6
	Female	182	47.2	200	51.4
Age of children in months	6-8	52	13.5	56	14.4
	9-23	334	86.5	333	85.6
Birth order	First to third	214	55.4	205	52.7
	Fourth & above	172	46.6	184	47.3
Number of U-5 children in the household	One	274	71.0	293	75.3
	Two and more	112	29.0	96	24.7
Family size	<=5	215	55.7	209	53.7
	>5	171	44.3	180	46.3
Age of the respondent in years	<=24	84	21.8	84	21.6
	25-34	180	46.6	179	46.0
	>=35	122	31.6	126	32.4
Marital status	Married	370	95.9	374	96.1
	Other (*)	16	4.1	15	3.8
Educational status	Can’t read &write	229	59.3	247	63.5
	Only read & Write	83	21.5	87	22.4
	Primary school (1-8)	41	10.6	29	7.5
	Secondary school (9-12)	23	6.0	12	3.1
	College/ university	10	2.6	14	3.6
Husband educational status	Can’t read and write	141	38.0	147	39.1
	Only Read and Write	138	37.2	160	42.6
	Primary school (1-8)	44	11.9	28	7.4
	Secondary school (9-12)	39	10.5	31	8.2
	College/ university	9	2.4	10	2.7
Husband occupation	Farmer	339	91.4	330	87.8
	Others (**)	32	8.6	46	12.2
Maternal involvement on decision	Yes	189	49	189	48.6
	No	197	51	200	51.4
Household Wealth status	Richest	66	17.1	69	17.7
	Rich	77	19.9	102	26.2
	Middle	74	19.2	82	21.1
	Poorer	84	21.8	69	17.7
	Poorest	85	22.0	67	17.2
Household food insecurity status	Food secure	279	72.3	289	74.3
	Mildly food insecure	94	24.4	69	17.7
	Moderately food insecure	13	3.4	31	8.0

*Single, Divorced, and widowed; ** merchant and daily worker

### Maternal and child health service characteristics

The preset study showed that 310 (79.7%) and 267 (69.2%) of the mothers from users and non-users gave birth at health facilities for their previous pregnancy, respectively. Less than half of mothers, 184 (47.3%) and 192(49.7%) from users and non-users had PNC follow-up for the study child, respectively. Only one hundred (28%) and 83 (21.5%) children’s weights had been measured every month at GMP sessions by users and non-users, respectively (Table 2).

**Table 2:** Maternal and child care characteristics of the respondents from users and non-users of North Mecha District, Northwest, Ethiopia, 2022 (n1= 389, n2 = 386)

Characteristics		Irrigation			
		Non-users (n2=386)		Users (n1=389)	
		Frequency	%	Frequency	%
ANC follow up	Fourth and above	61	15.8	153	39.3
	One to three	315	81.6	179	46.0
	Not visit	10	2.6	57	20.3
Place of delivery	Home	119	30.8	79	20.3
	Health facility	267	69.2	310	79.7
PNC follow up	Yes	192	49.7	184	47.3
	No	194	50.3	205	52.7
When started PNC?	Within 1-2 day	98	51.0	84	45.7
	Within 3-6 days	45	23.4	49	26.6
	After 7 days	49	25.5	51	27.7
Pre-lacteal feeding	No	359	93.0	362	93.1
	Yes	27	7.0	27	6.9
Initiation of BF	Within 1 h	67	17.4	102	26.2
	After 1 h	319	82.6	287	73.8
Complementary feeding initiated	Before 6 months	39	10.1	33	8.5
	At 6 months	233	60.4	232	59.6
	After 6 months	101	26.2	112	28.8
	Not started	13	3.4	12	3.1
Ever receive vaccines?	Yes	344	89.1	386	99.2
	No	42	10.9	3	0.8
Vaccination status	Up to date	57	16.6	67	17.4
	Fully	287	83.4	319	82.6
Vit-A Supplementation	Yes	245	63.5	247	63.5
	No	141	36.5	142	36.5
Got GMP service?	Yes	83	21.5	109	28.0
	No	303	78.5	280	72.0
Illness in the last 2 weeks	Yes	72	18.7	80	20.6
	No	314	81.3	309	79.4
Type of the illness	Fever	18	25.0	18	22.5
	Diarrhea	33	45.8	43	53.8
	Cough	21	29.2	19	23.7
Maternal knowledge of IYCF Practice	Good	288	74.6	292	75.1
	Poor	98	25.4	97	24.9

### Minimum dietary diversity and minimum meal frequency

**Fig. 2:**
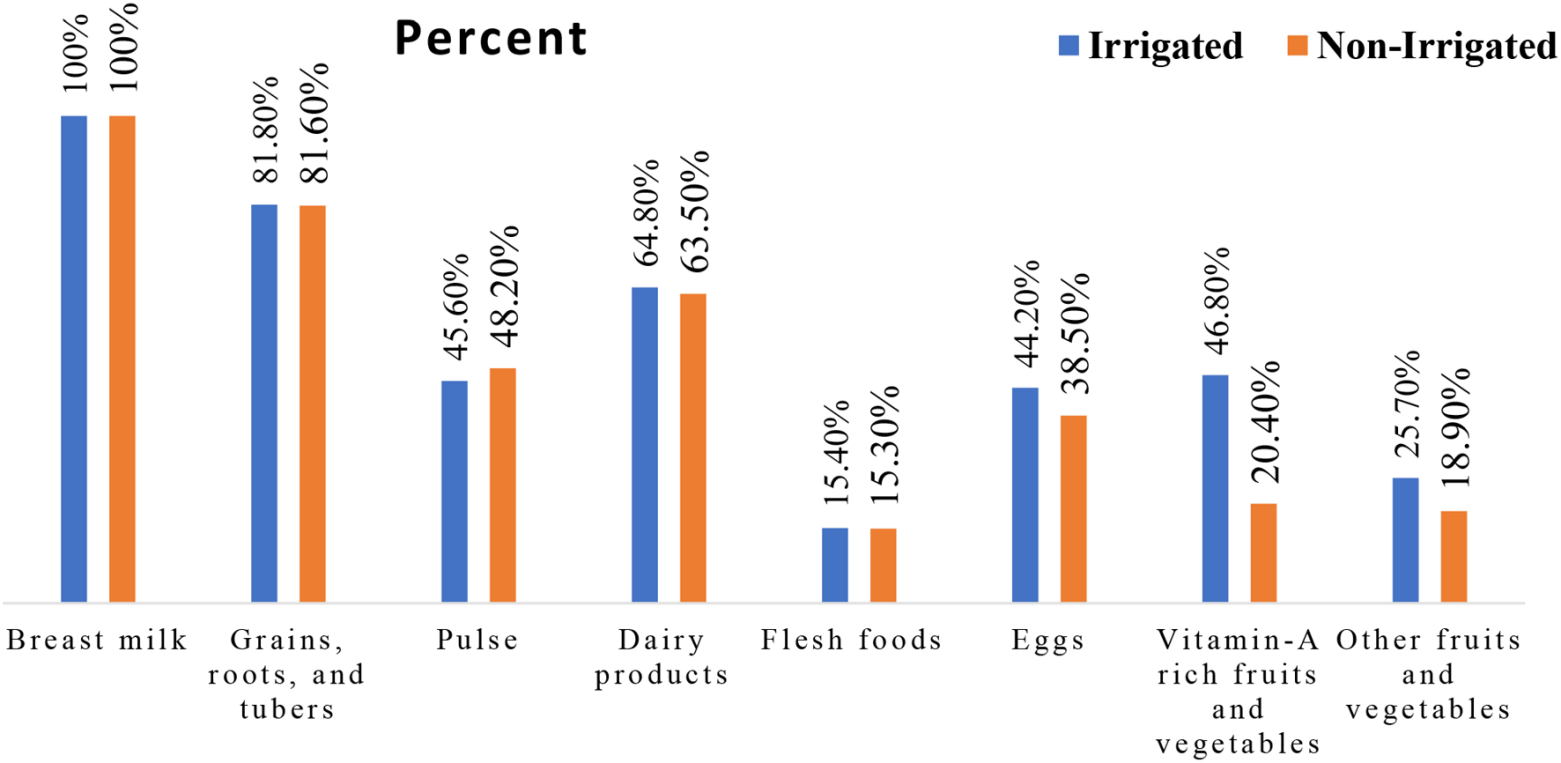
Percentage of each food group consumption by children aged 6–23 months 24-hours before the survey from users(n1) and non-users (2) of North Mecha District, Northwest, Ethiopia, 2022 (n1 = 389, n2 = 386)

**Fig. 3:**
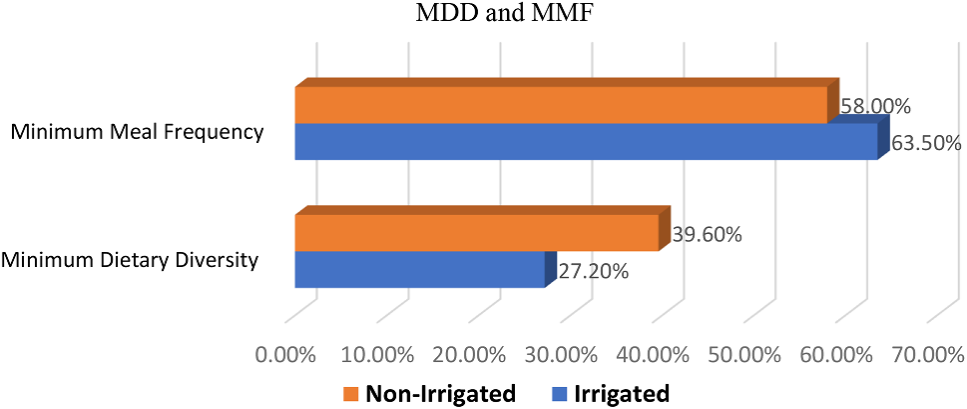
Percentage of MDD and MMF consumption by children aged 6–23 months 24-hours before the survey from users(n1) and non-users (2) of North Mecha District, Northwest, Ethiopia, 2022 (n1 = 389, n2 = 386)

When we examine the food group consumption of the study subjects, all of them, both users and non-users consumed breast milk in the last twenty-four hours prior to the study (Fig. 2). The proportion of children who received the recommended minimum dietary diversity was 39.6% among users and 27.2% among non-users. The proportion of children who received the recommended meal frequency was 63.5% and 58.0% among users and non-users, respectively (Fig. 3).

### Comparison of minimum acceptable diet among users and non-users

**Fig. 4:**
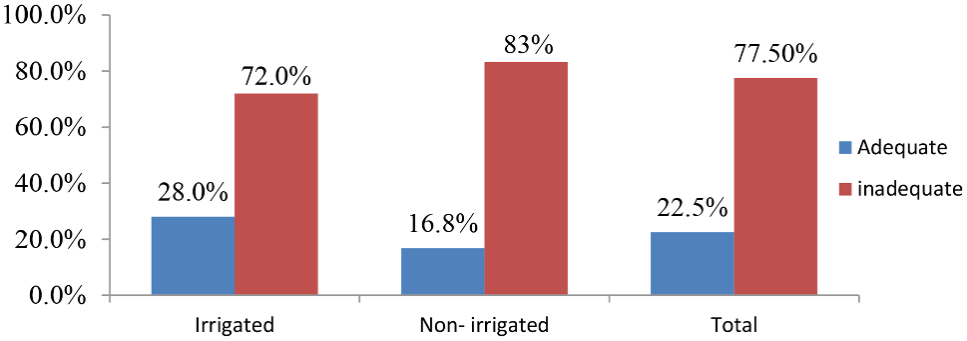
Magnitude of minimum acceptable diet practice among 6–23 months children from users and non-users of North Mecha District, Northwest, Ethiopia, 2022 (n1 = 389, n2 = 386)

There was a significant difference in magnitudes of MAD among users and non-users (X^2^ = 13.912, *P* <.001). The overall magnitude of recommended minimum acceptable diet practice was 22.5%, and the MAD practice among users was 28.0% (95% CI: 23.7, 32.4), and for non-user was 16.8% (95% CI: 13.2, 20.7) (Fig. 4).

### Factors associated with minimum acceptable diet practice

#### Factors associated with MAD practice among users

Bivariate and multivariable logistic regression analyses were done to establish an association between the independent variables and the outcome variables. During bivariate logistic regression: analysis occupation of husband, involvement in the decision, family size, ANC, Place of birth, pre-lacteal feeding, timely initiation of BF feeding, bottle feeding, child illness in the last two weeks, and households’ food insecurity were candidates variables for multivariable logistic regression analysis at *P* <.2. Whereas, the multivariable logistic regression analysis revealed that, three variables namely involvement in decisions, timely initiation of breastfeeding, and child illness in the last two weeks were found to be significantly associated with MAD in children among users.

Children born from mothers who were not involved in the decisions were 4.37 times more likely to have an inadequate minimum acceptable diet practice than children born from mothers involved in household decisions [AOR = 4.37, 95% CI: (2.05,9.33)]. Children who started breastfeeding after one hour of delivery were 5.29 times more likely to practice an inadequate minimum acceptable diet than those children who started breastfeeding within one hour after delivery [AOR = 5.29, 95% CI: ( 2.393,11.672)]. Children who had the illness in the last 2 weeks were 4.10 times more likely to have an inadequate minimum acceptable diet practice than those children who hadn’t a history of illness in the last two weeks [AOR = 4.10, 95%CI: ( 1.48,11.38)] (Table 3).

**Table 3:** Associated factors of MAD 6 to 23 months children from users of North Mecha District, Northwest Ethiopia, 2022 (*n*= 389)

Characteristics	Minimum acceptable diet (MAD) among users (*n*= 389)			
	Inadequate n (%)	Adequate n (%)	COR (95% CI)	AOR (95% CI)
Husband’s occupation				
Farmer	242(73.3)	88(26.7)	1	1
Others (**)	28(60.9)	18(39.1)	0.57(0.30, 1.07)	1.39(0.38, 5.08)
Involved on decision				
No	171(85.5)	29(14.5)	4.33(2.66, 7.05)	4.37(2.05, 9.32) *
Yes	109(57.7)	80(42.3)	1	1
Family size				
<=5	138(66.0)	71(34.0)	1	1
>5	142(78.9)	38(21.1)	1.92(1.22, 3.04)	1.55(0.64, 3.76)
ANC follow up				
No	51(89.5)	6(10.5)	1	1
1-3	132(73.7)	47(26.3)	0.33(0.13, 0.82)	0.56(0.10, 2.98)
>=4	97(63.4)	56(36.6)	0.20(0.08, 0.51)	0.46(0.09, 2.37)
Place of delivery				
Home	69(87.3)	10(12.7)	1	1
Health facility	211(68.1)	99(31.9)	0.31(0.15, 0.63)	0.47(0.12, 1.86)
Pre-lacteal feeding				
No	266(73.5)	96(26.5)	1	1
Yes	14(51.9)	13(48.1)	0.39(0.18, 0.86)	0.63(0.15, 2.59)
Initiation of BF				
Within 1 h	52(51.0)	50(49.0)	1	1
After 1 h	228(79.4)	59(20.6)	3.71(2.29, 6.01)	5.29(2.39, 11.67) *
Bottle-feeding				
No	252(75.2)	83(24.8)	1	1
Yes	28(51.9)	26(48.1)	0.36(0.20, 0.64)	0.45(0.14, 1.49)
Illness in the last 2wk				
Yes	69(95.1)	11(13.8)	2.91(1.48, 5.75)	4.10(1.48, 11.38) *
No	211(68.3)	98(31.7)	1	1
HH Food Insecure				
Food secure	199(68.9)	90(31.1)	1	1
Food Insecure	81(81)	19(19)	1.93(1.10, 3.37)	1.63(0.56,4.73 )

*Note* *Indicates significant at *P*<.001 ** Merchant and daily worker

*Abbreviation* COR, Crude odd ratio, and AOR, adjusted odds ratio. HH, Household

#### Factors Associated with MAD practice among non-users

During the bivariate logistic regression analysis involved in the decisions, Place of delivery, PNC follow-up, pre-lacteal feeding, initiation of BF feeding, vitamin A supplementation, GMP service utilization, and child illness in the last two weeks were found as candidate variables for multivariable logistic regression analysis at *P* <.2. However, on multivariable logistic regression analysis, four variables, namely involvement in the decision, place of delivery, PNC follow-up, and GMP service utilization, were found to be significantly associated with MAD in children from non-users.

Children born from mothers who were not involved in the decisions were 4.71 times more likely to practice an inadequate minimum acceptable diet than children born from mothers involved in household decisions [AOR = 4.71, 95% CI: (2.28, 9.75)]. Children delivered at home were 2.51 times more likely to have an inadequate minimum acceptable diet practice when compared to children delivered at a health institution [AOR = 2.51, 95% CI: (1.14, 5.55)].

Children born from mothers who reported not attending PNC follow-up were 3.01 times more likely to practice inadequate MAD than children born from those who reported attending PNC follow-up [AOR = 3.01, 95% CI: (1.57, 5.77)]. Similarly, those mothers who reported not attending GMP service to their children were 4.64 times more likely to practice inadequate MAD than their counters [AOR = 4.64, 95%CI: (2.40, 8.95)] (Table 4).

**Table 4:** Associated factors of MAD 6 to 23 months children among non-users of North Mecha District, Northwest Ethiopia, 2022 (*n*= 386)

Characteristics	Minimum acceptable diet (MAD) among non-users (*n*= 386)			
	Inadequate n (%)	Adequate n (%)	COR (95% CI)	AOR (95% CI)
Involved on decision				
Yes	136(42.4)	53(81.5)	1	1
No	185(57.6)	12(18.5)	6.01(3.09, 11.68)	4.71(2.28, 9.75) **
Place of delivery				
Home	109(34.0)	10(15.4)	2.83(1.39, 5.77)	2.51(1.14, 5.55) *
Health facility	212(66.0)	55(84.6)	1	1
Had PNC follow up				
Yes	152(47.4)	40(61.5)	1	1
No	169(52.6)	25(38.5)	1.78(1.03, 3.07)	3.01(1.57, 5.77) **
Pre-lacteal feeding				
Yes	20(6.2)	7(10.8)	1	1
No	301(93.8)	58(89.2)	0.55(0.22,1.36)	0.780(0.27, 2.26)
Initiation of BF				
Within 1 h	51(15.9)	16(24.6)	1	1
After 1 h	270(84.1)	49(75.4)	1.73(0.91,3.27)	1.90(0.87, 4.18)
Vi-A Supplementation				
Yes	213(66.4)	32(49.2)	2.03(1.19, 3.49)	1.57(0.83, 2.98)
No	108(33.6)	33(50.8)	1	1
Got GMP service				
Yes	48(15.0)	35(53.8)	1	1
No	273(85.0)	30(46.2)	6.64(3.73, 11.81)	4.64(2.40, 8.95)**
Illness in the last 2wks				
Yes	65(20.2)	7(10.8)	2.10(0.92, 4.83)	1.76(0.70, 4.41)
No	256(79.8)	58(89.2)	1	1

*Note* *Indicates significant at * *P*<.05 **Indicates significant at *P*<.001

*Abbreviation* COR, Crude odd ratio, and AOR, adjusted odds rat

## Discussion

The findings of this study indicated that the prevalence of adequate MAD practice for children aged 6–23 months among households with irrigation land users and non-users was 28% (95% CI: 23.7, 32.4) and 16.8% (95% CI: 13.2, 20.7), respectively. This indicates that MAD practice of under two children in irrigated users are significantly higher than non-users (X^2^ = 13.912, *P* <.001). This significant variation is happening due to the irrigation scheme, since irrigation can encourage crop diversification and the production of more diverse foods for household consumption. The mothers in the irrigated area can easily access a variety of food items as they have the opportunity to grow vegetables and fruits in the dry season by irrigation than in non-irrigated areas. This is an advantage to enhancing minimum dietary diversity, minimum meal frequency, and improving minimum acceptable diet practice. For instance, evidence shows that agricultural intervention like irrigation are a best practice to improve complementary feeding among children [34]. Another study has also indicated that access to irrigation farmland enhances crop diversity, which in turn leads to improved children’s dietary diversity and frequency [35, 36].

The preset study showed that the magnitude of an adequate minimum acceptable diet among irrigation users was 28%, which was higher compared with studies conducted in Goncha district, Northwest Ethiopia, (12.6%) [37], North Shoa, Oromia Region (13.3%) [38], and from the national report (7%) [32]. It was also higher than the study conducted in a number of African countries, including Kenya, 21.8% [39], Burkina Faso, 13% [40], and Ghana, 17% [41]. However, the current finding is lower than studies conducted in Addis Ababa 74.6% [42], Nepal 44.3% [43], and China 49.0% [44]. This is probably due to the difference in the socio-economic, socio-demographic, and socio-ecologic characteristics of the study subjects; for instance, people livening in Addis Ababa are higher than those in our study area in terms of socio-economic status.

In the current study, 16.8% of children aged 6–23 months received an adequate minimum acceptable diet from children form non- irrigation users. The finding is higher than previous reports, including; Dembech district (8.6%) [45] and the EDHS report of 2016 (7%) [32]. The variation might be due to the difference in study periods. For example, the study in Dembech district was conducted during the fasting period of orthodox religion followers during which feeding habits might be reduced either in food diversity, especially animal source foods or meal frequency which estimates the finding when compared to other periods. With regard to the EDHS report, the difference might be due to the EDHS study being conducted countrywide in culturally different populations, which may underrate child feeding practices, while the current study was conducted on an almost culturally homogenous population with similar feeding practices. However, the current finding is less than studies conducted in Debre Berhan (31.6%) [7], Mareka District, Southern Ethiopia, (35.5%) [28]. This is probably due to the difference in the socio-economic, socio-demographic, and study periods; the current study was conducted in rural communities, whereas Debre Berhan and Mareka district studies were conducted in urban communities which are higher than those in our study area in terms of socio-economic status. As communities in rural areas are less likely to feed a minimum acceptable diet than people residing in urban areas [32]. Also, the difference might be due to the fact that more non-educated mothers participated in this study; on the contrary, a higher number of educated participants were included in the above study. This finding is also higher than the study conducted in Nigeria (9.2%) [46], and Malawi (12%) [47], and less than the study conducted in Kenya (21.8%) [39], and Ghana (17%) [41]. The variation might be due to differences in socio-demographic characteristics and study period.

A significant association was observed between children born from mothers who were involved in the decision and the minimum acceptable diet practice for both users and non-users. The magnitude of inadequate MAD practice was significantly higher among those who were not involved for in the decision as compared to those who had been involved. The possible explanation might be that mothers who were involved in the decision can get free time to feed their children and can easily purchase foods that are not available in the household. In addition to this, mothers who were involved in income-generating irrigation activities and their control of income from irrigation had a greater impact on increasing the child MAD practice of the households. This finding was supported by previous studies conducted in Denbecha district in northwest Ethiopia [45].

In this study, among users’ children who had an illness in the last two weeks, there was a significant association with an inadequate minimum acceptable diet practice. In this study, children among users who had an illness in the last two weeks were more likely to have an inadequate minimum acceptable diet than those who hadn’t had a history of illness in the last two weeks. This result was supported by the study conducted in Nepal and Debre Berhan town [7, 43]. This is because illness reduces children’s appetite, dietary intake, and nutrient absorption, leading to inadequate MAD.

This study showed that among non-users, place of delivery was significantly associated with child MAD practice. Children delivered at home were more likely to have an inadequate minimum acceptable diet practice when compared to children at health institution. The possible description might be due to the fact that during institution delivery, health professional counseling on appropriate child feeding after delivery to a health facility increases mothers’ awareness of the practice a minimum acceptable diet; hence, mothers’ awareness of appropriate child feeding practices who got it from health professionals have had better child feeding practices than their counterparts. Furthermore, it might enhance information about PNC follow-up and child health services, increase maternal knowledge, and promote MAD practice. This finding is supported by studies conducted in the Denbecha district in Northwest Ethiopia, Mareka District Southern Ethiopia, and Nepal [28, 43, 45].

In this study, PNC follow-up was associated with MAD practice among non-users of 6-23-month-old children. Children born from mothers who reported not attending PNC follow-up were more likely to practice inadequate MAD than children born from those who reported attending PNC follow-up. This could be since nutritional advice and counseling by health workers might not only educate mothers but also avoid traditional beliefs that might inhibit child feeding practices. Furthermore, it might be the strength of health extension worker implementation in maternal health service packages, including postnatal services. This finding is supported by the findings from in Democratic Republic of Congo, Nepal, and North Shoa, Oromia Region, Ethiopia [38, 43, 48].

Another important variable found to be associated with child MAD among non-users was GMP service utilization. Those mothers who reported not attending GMP services for their children were more likely to practice inadequate MAD than their counters. This might be as the HEWs monitor the weight of children using the standard GMP charts, and they will provide nutrition counseling to mothers or caregivers of children; as a result, there might be an improvement in child feeding practices [49]. These activities also provide an opportunity for early recognition of signs of undernutrition, and any illness and to manage them accordingly. This flinging is also supported by studies conducted in northern and southern parts of Ethiopia, which indicated that there is more likely for meeting MAD in those children attending regular GMP service utilization at each health post level [28, 37].

## Conclusion and recommendation

The overall prevalence of minimum acceptable diet practice was low compared with national and WHO recommendations. But the practice was much higher among irrigated users than among non-irrigated users. Involvement in a decision, place of delivery, PNC, and GMP are independent predictors of MAD in children from non-irrigated households. The finding reveals that a significant association was observed between irrigation status and MAD practice in the study area. The study suggested that expanding access to irrigation for households may be the best approach to improving child nutrition.

## Data Availability

The datasets used and/or analyzed during the current study available from the corresponding author on reasonable request. If further is required, you can communicate me though my email: seoumer@yhoo.com.
